# Anal Itchings

**Published:** 1879-10

**Authors:** 


### ANAL ITCHINGS.

THIS is a malady which is never referred to, except in professional works, and yet it is an ailment which gives an incredible amount of annoyance, coming on as it does on retiring to bed, and continuing nightly for many years, making sleep impracticable, sometimes for many hours together. It is sometimes a dyspeptic symptom, at others, it arises from a multitude of small worms at the parts. As an unprofessional man is not likely to know the real cause, and yet may not like to ask for advice, strict cleanliness and frequent ablutions are essential; then regulate the diet, living mainly on cold bread, fruits, and fresh meats. But for instantaneous relief, inject a teaspoonful of camphor water, or dip the fore-finger in the water, and apply it. One or two applications are often sufficient. Or apply Ne[illegible]at.ons

